# CD8^+^ T Cells in Association with SCD1 Regulate Cervical Cancer and Tumour Microenvironment Correlation Research

**DOI:** 10.1007/s43032-026-02070-2

**Published:** 2026-04-22

**Authors:** Qi Bao, Zihan Wang, Guanghua Gu, Wei Wang, Shuangyang Qin, Shihui Zhang, Weipei Zhu

**Affiliations:** 1 Department of Obstetrics and Gynecology, Liyang People’s Hospital, Liyang, Jiangsu Province China; 2 Kangda College of Nanjing Medical University, Lianyungang, Jiangsu Province China; 3 Department of Obstetrics and Gynecology, Second Affiliated Hospital of Soochow University, No. 1055 Sangxiang Road, Suzhou, 215100 Jiangsu Province People’s Republic of China; 4 Department of Obstetrics and Gynecology, Zhenjiang First People’s Hospital, Zhenjiang, 212002 Jiangsu Province China

**Keywords:** SCD1, Ferroptosis, Cervical cancer, ROS, CD8^+^ T

## Abstract

Objective To explore new strategies for treating cervical cancer and tumour microenvironment correlation. Methods Clinical significance and prognostic impact of SCD (Stearoyl-CoA Desaturase ) in cervical cancer through bioinformatics analysis of the GTEx project and the TCGA database. Immunohistochemical analysis of SCD1 expression in clinical cervical cancer tissues and normal cervical tissues. CCK-8 assay, cell cycle, transwell assay, western blotting, transmission electron microscopy, reactive oxygen species (ROS) detection, lipid peroxidation detection, and ferroptosis analysis were performed to reveal the role and underlying mechanisms in cervical cancer cell line (HeLa & CaSki). Results SCD1 is highly expressed in clinical cervical cancer tissues, especially in advanced cancer. Knockdown SCD1 inhibits proliferation and migration in cervical cancer cells. Knockdown SCD1 induces ferroptosis in cervical cancer cells by upregulating ROS, Fe^2+^, lipid peroxidation, ferroptosis indicator (Acyl-CoA synthetase long chain family member 4, ACSL4) and downregulating of ferroptosis indicator (Glutathione Peroxidase 4, GPX4), mitochondrial membrane potential (MMP). Transmission electron microscopy revealed morphological changes in mitochondria after knockdowning SCD1. 72 cervical cancer cases (63 stage I-II and 9 stage III-IV) were retrospectively analysed and confirmed statistical significant difference in expression of SCD1 in early and advanced cervical cancer. CD8^+^ T cell expression was significantly reduced in cancer tissues with high SCD1 expression. There was significant heterogeneity in CD8^+^ T cell expression in peripheral blood and cancer tissues. Conclusion This study suggested that knockdown SCD1 acts as a novel ferroptosis-inducing agent in cervical cancer cells, and the relationship between SCD1 and CD8⁺ T cells is inversal.

**Supplementary Information:**

The online version contains supplementary material available at 10.1007/s43032-026-02070-2.

## Background

Cervical cancer is one of the most common malignancies in women, second only to breast cancer. With the popularisation and improvement of cervical cancer screening and treatment methods, many patients can be diagnosed and treated in time, but the prognosis for patients with advanced cervical cancer is generally poor, posing a serious threat to women’s physical and mental health. Currently, surgery and radiotherapy is the main clinical treatment for cervical cancer. However, due to the characteristics of cervical cancer itself and the abnormal expression of certain genes, the cancerous foci tend to be insensitive to conventional radiotherapy, severely weakening the therapeutic effect. Current cervical cancer treatments are not only inadequate to treat metastatic cervical cancer, but are also considered cytotoxic in some patients, causing serious side effects that can affect women’s life quality [1]. Therefore, from the perspective of the pathogenesis of cervical cancer, it is extremely important to discover appropriate and effective biomarkers related to diagnosis, treatment and prognosis, and then to identify new and effective therapeutic targets for treatments of cervical cancer.

SCD is the rate-limiting enzyme that catalyses the conversion of saturated fatty acids to monounsaturated fatty acids. Tesfay [2] et al. found that SCD is highly expressed in ovarian cancer tissue, ovarian cancer cell lines and a mouse model of in situ ovarian cancer cell transplantation. There are two isoforms of human SCD (SCD1 and SCD5), with SCD1 is more prevalent [3]. Current studies do not fully reveal the exact mechanism of SCD1 and cellular ferroptosis. Lipid analysis of tumour cells showed a gradual increase in the conversion of saturated fatty acids to monounsaturated fatty acids, whereas when the SCD1 inhibitor CoQ10 was added to ovarian cancer cell lines and mouse models, SCD1 expression decreased and upregulation of cellular ferroptosis was observed, suggesting that the inhibition of SCD1 enhances the ferroptosis pathway in cancer cells and exerts anti-tumour effects. Carbone [4] observed that SCD1 inhibitors induced apoptosis and ferroptosis in ovarian cancer cells in vitro and in vivo, and in particular, the combination of SCD1 inhibitors and ferroptosis inducers significantly reduced the size of ovarian tumours in mice, suggesting that this combination therapy may be effective in the treatment of ovarian cancer. SCD1 causes fatty acid desaturation and fatty acid binding protein 4 enhances binding to fatty acids, both of which together promote cancer cells from oxidative stress-induced ferroptosis. The essence of ferroptosis is the attack of intracellular biomolecules by lipid reactive oxygen species generated by membrane lipid peroxidation (desaturation), leading to cell death [5]. Studies have reported that SCD is highly expressed in a variety of tumour tissues and promotes the proliferation and migration of tumour cells, which is associated with to tumourigenesis and development [6]. However, the function of SCD1 in cervical cancer is still unknown. At present, the regulatory role of SCD1 in cervical cancer has been documented in only a very limited number of studies, e.g. IGF2BP3 enhances lipid metabolism in cervical cancer by upregulating the expression of SCD [7]. Promotes cervical cancer angiogenesis by upregulating SCD to activate ERK pathway [8]. To determine the exact role of SCD1 in cervical tumorigenesis, this study demonstrated that SCD1 modulates proliferation and migration of cervical cancer cells via the mitochondrial pathway while inducing ferroptosis. The unique inverse relationship between SCD1 and CD8⁺ T cells has not been previously reported in cervical cancer. The findings provide a new theoretical basis for the pathogenesis of cervical cancer and imply that SCD1 could be a potential target for its treatment.

A long-standing problem in cancer biology is that oxidised tumours ferment most of the glucose they consume, rather than oxidising it in the mitochondria. This phenomenon is known as the ‘Warburg effect’ [9]. Oxidative stress is an imbalance of harmful reactive oxygen species (ROS) and neutralisation of antioxidant mechanisms. If left unchecked, the deleterious effects of oxidative stress can lead to damage to DNA, proteins and cell membranes, ultimately leading to cell death. Tumours are highly proliferative and therefore produce high levels of mitochondrial ROS, to compensate for this and to maintain redox homeostasis, cancer cells utilise protective antioxidant pathways, which are further amplified in drug-resistant tumours [10]. Chemotherapy and radiotherapy combined with DNA damage produce high levels of oxidative stress in tumours [11]. Oxidative stress causes damage to DNA, proteins and lipids and ultimately triggers cell death. The nature of cellular iron death is for iron-dependent membrane lipid peroxidation (desaturation) to generate lipid reactive oxygen species that attack intracellular biomolecules, disrupting the intracellular redox balance and triggering cell death [5]. Ferroptosis is now known to be associated with pathological cell death in a variety of degenerative diseases (e.g. Alzheimer’s disease, Huntington’s disease and Parkinson’s disease), cancer, stroke, cerebral haemorrhage, traumatic brain injury, local ischaemia-reperfusion injury and renal degeneration. It is becoming clear that this form of cell death may be closely related to the development of many diseases and it is realised that ferroptosis can be used as a basis for the development of a wide range of pharmacological treatments [12]. Therefore, how to affect ferroptosis through targeted metabolic regulation to improve the therapeutic efficacy of cervical cancer has become one of the hot issues in current research.Therefore, how to affect ferroptosis through targeted metabolic regulation to improve the therapeutic efficacy of cervical cancer has become one of the hot issues in current research.

Tumours and the environment are interdependent and mutually reinforcing, as well as antagonistic and struggling against each other. With the progress of tumour cytology and molecular biology in recent years, we have gained a deeper understanding of the interrelationship between tumours and the microenvironment. This is not only important for understanding the occurrence, development and metastasis of tumours, but also plays an indispensable role in the diagnosis, prevention and prognosis of tumours. Characteristics of the tumour microenvironment mainly include: hypoxia, acidification, interstitial hypertension, vascular hyperpermeability, inflammatory response, immunosuppression, and other factors [12] Immune cells are classified as lymphocytes, monocytes and granulocytes [13]. Lymphocytes are classified into: T cells, B cells, NK cells and macrophages.T cells are further classified into: regulatory T cells, helper T cells and cytotoxic T cells. Killer T cells (CTL cells), often referred to as CD8^+^ T cells, are a key component of the adaptive immune system and play an important role in the immune system’s defence against pathogens such as viruses, bacteria and tumours [14]. They function by killing infected or cancerous cells through direct or indirect methods. Activation of CD8^+^ T cells in cancer involves an initial activation phase in the lymph nodes and subsequent effector differentiation within the tumour.CD8^+^ T cells are an important component of the adaptive immune response to cancer, and infiltration of these cells into the tumour predicts patient survival and response to checkpoint therapy. The fact that some patients have a large T-cell infiltrate, while others have virtually none, requires a better understanding of the underlying mechanism that controls the T-cell response to cancer, which is currently unclear. Recent studies [14] are moving towards identifying strategies to release T cell activity by targeting T cell metabolism. Efforts are also being made to improve the efficacy of immune checkpoint blockade and adoptive cell transfer therapies.

Knockdown of SCD1 induces cervical cancer cells to die in an ferroptosis mode whether there is a correlation with the tumour microenvironment, which deserves to be explored further.

## Materials and Methods

### Cell Lines and Cell Culture

HeLa cells were cultured in our laboratory. All cell lines were grown in Dulbecco’s modified Eagle’s medium (DMEM) supplemented with 10% fetal bovine serum (FBS) and incubated at 37 °C in 5% CO2 environment. Comprehensive testing confirmed that all cell lines were free of mycoplasma contamination. Three technical and biological replicates per experiment at least.

### RNA Isolation and Real-Time Quantitative PCR

Total RNA was extracted from fresh tissues with Trizol reagents (Invitrogen, Carlsbad, CA, USA) following the manufacturer’s instructions.mRNAs were reverse transcribed to generate complementary DNA (cDNA) using oligo (dT) primers. GAPDH was considered as the endogenous control. Quantitative reverse transcriptase-polymerase chain reaction was performed using LightCycler Fast Start DNA Master SYBR Green I on a LightCycler (Roche Diagnostics GmbH, Mannheim, Germany) system. The comparative cycle threshold method (ΔΔCt) was used to quantify the relative expression of each gene. The samples were loaded in triplicate, and the results of each sample were normalized to GAPDH. The primers used in the present study were listed in Supplementary Figure 2.

## Antibodies and Reagents

The following antibodies employed in this research: anti-GPX4 (SC-166570, Santa Cruze); anti-ACSL4 (SC-271800, Santa Cruze); anti-β-actin (T0022, Affinity), anti-SCD1 (clone EPR21963, #ab236868, Abcam), anti-α-Tublin (ab7291, Abcam).

## EdU Assay

CaSki cells were plated in 96-well plate for 48 h, cell proliferation was detected using the incorporation of 5-ethynyl-2′-deoxyuridine (EdU) with the EdU cell proliferation assay kit (Guangzhou RiboBio Co., Ltd. Guangzhou, China). Briefly, the cells were incubated with 50µM EdU for 2 h before fixation, permeabilization and EdU staining according to the manufacturer’s protocol. The proportion of EdU positive cells was determined by flow cytometry (Becton Dickinson, San Jose, CA, USA).

## Cell Proliferation Assay

A Cell Counting Kit-8 (CCK-8; Yeasen, Shanghai) was utilized for executing cell proliferation assessments in accordance with the manufacturer’s protocol. The experiments were performed in quadruplicate. All experiments were repeated at least three times.

## Cell Cycle Assay

In summary, a total of 106 cells were cultured in 6-well plates. After siRNA transfection, the adherent cells were washed once with PBS, trypsinized, and collected by centrifugation at 400 × g for 5 min. The cells (106 cells per sample) were fixed in 4 ml of cooled 70% ethanol at − 20 °C overnight. After centrifugation at 1000 × g for 10 min, cell pellets were incubated with 0.5 ml of PBS containing 100 µg/ml RNase (Invitrogen) and 5 µg/ml propidium iodide (Sigma-Aldrich) at room temperature for 30 min. Cell cycle distribution was analyzed by measuring DNA content using flow cytometry. The experiment was repeated 3 times and each contained at leas three biological replicates.

### Transwell Assay

Migration analyses were performed separately using Corning permeable plates according to the manufacturer’s protocol. For migration, cells (0.5 × 10^5^ in 100 µL of serum-free DMEM) were introducted into the upper chambers, while the lower chamber of each well contained 700 µL of DMEM supplemented with 10% FBS. The incubation process took place in an incubator at 37 °C in 5% CO_2_ for 16 h. Cells that migrated to the bottom of the upper chamber membrane were stained with crystal violet.

## Western Blotting Analysis

Cultured cells were lysed in lysis buffer supplemented with protease and phosphatase inhibitors. Protein concentrations of the lysates were determined by the BCA protein assay system (Beyotime). Equal amounts of protein were separated by 10% SDS-PAGE, transferred to PVDF membrane (Millipore, Billerica, MA).

Detection of mitochondrial ROS, Fe^2+^ and mitochondrial membrane potential (MMP).

Cells were then incubated with 5 µM DCFH-DA (S0033s, Beyotime) or 5 µM FerroOrange (M36008, Thermo Fisher Scientific) in HBSS for the detection of ROS, Fe^2+^ (F374,Dojindo), respectively. MMP was determined by incubating cells with 2 µM JC-1 (C2003S, Beyotime) fluorescent probe, which yields green fluorescence at low concentrations while lights up in red when it accumulates in the mitochondria at high concentrations. Twenty minutes after incubation, cells were washed 3 times with HBSS buffer and subject to flow cytometry.

## Lipid Peroxidation Assay

Cultured cells were incubated for 20 min with 2 µM BODIPY^®^ 581/591 C11 (D3861, Invitrogen). After washing with PBS, cells were washed 3 times with HBSS buffer and subject to flow cytometry, FITC.

### Transmission Electron Microscopy (TEM)

The samples was fixed in 2.5% glutaraldehyde (pH 7.4) for 2 h. After washed three times with 0.1 M phosphate buffer (pH 7.2) and fixed in 1% osmic acid at 4℃ for 2 h. Then the samples was gradient dehydrated in a graded series of ethanol. Subsequently, the samples was embedded in Epon-Araldite resin for penetration and placed in a mold for polymerization. After the semi thin section was used for positioning, the ultrathin section was made and collected for microstructure analysis.Followed the counterstaining of 3% uranyl acetate and 2.7% lead citrate.Then observed with a transmission electron microscope (JEM1400, Jeol).

### Immunohistochemistry

Retrospectively analysed the cervical tissues of 72 cervical cancer cases and 63 cervical tissues of cases who underwent total hysterectomy for benign uterine diseases during the same period in the Second Affiliated Hospital of Soochow University [Suzhou, 12 Mar, 2024, Ethics Number EC2024(238)] and the Liyang People’s Hospital (Liyang, 30 Nov, 2023, Ethics Number 2023021) of from December 2022 to November 2024. We used retrospective samples in the immunohistochemistry study, so we applied for a waiver of informed consent during the ethical examination. The tissue was taken and fixed inside 4% paraformaldehyde for 3–4 h. Perform dehydration, using alcohol and anhydrous ethanol in that order. Embed the tissue sample and fix it with paraffin. Slice and dewax to water. Antigen repair, inactivation, blocking, primary antibody (clone EPR21963, #ab236868, Abcam, dilution: 1:2000, incubation time over night at 4 °C) incubation, secondary antibody incubation, colour development, re-staining and sealing.

### Immunofluorescence Staining of SCD1 and CD8^+^T

Immunofluorescence staining was carried out as described previously. Briefly, cells grown on coverslips were fixed in 4% paraformaldehyde for 10 min. The cells were then permeabilized in 0.2% Triton X-100 for 10 min, and blocked in 10% normal goat serum overnight at 4 °C. The coverslips were incubated with antiphospho-SCD1 (SCD1, abcam, ab236868, ab19862) and CD8^+^T (abcam, ab245118, ab237709) antibody overnight at 4 °C, washed in PBS and incubated with TRITC-conjugated Goat anti-mouse secondary antibody (Jackson ImmunoResearch Laboratories, West Grove, PA, USA) for 1 h at room temperature. Cells were washed in PBS three times and counterstained with DAPI(Beyotime, C1006). Fluorescence images were captured under a fluorescence microscope.

### Statistical Analysis

Continuous variables such as age and fertility history were compared using Student’s t-test or Mann–Whitney U test depending on distribution normality assessed by the Shapiro–Wilk test. Difference was considered significant if the *p* value was less than 0.05.

## Results

1. **SCD (SCD1) is highly expressed in cervical cancer**,** has a poor prognosis and is significantly associated with the ferroptosis pathway.**

**Fig. 1:**
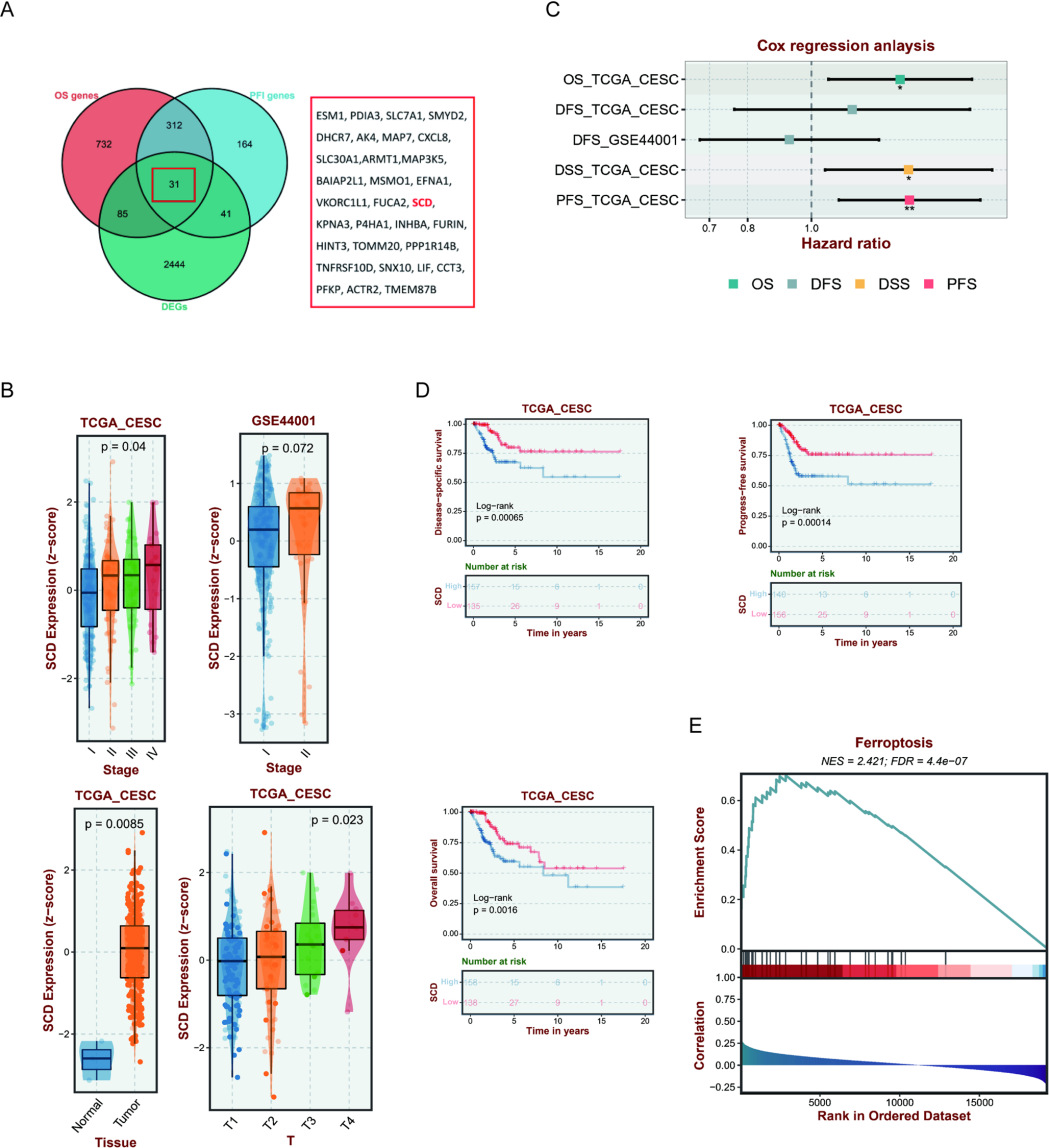
SCD (SCD1) is highly expressed in cervical cancer, has a poor prognosis and is significantly associated with the ferroptosis pathway (**A**) 31 potential oncogenes based on a list of prognosis-related genes with highly expressed genes in cervical cancer tumour tissue rom the TCGA database. (**B**) Relationship between SCD and clinical features. (**C**, **D**) Prognostic indications of SCD in patients with cervical cancer. Relative to patients with low-expression SCD, the high-expression SCD group had shorter overall survival time, disease-free survival interval, and progression-free survival. (**E**) GSEA analysis demonstrated a significant association of the ferroptosis pathway with high SCD expression

We collaboratively analysed the GTEx project with cervical cancer samples from the TCGA database and identified 31 potential oncogenes based on a list of prognosis-related genes with highly expressed genes in tumour tissue (Fig. 1A). Through literature search and analysis, we found that among the 31 potential oncogenes, the SCD (stearoyl coenzyme A desaturase) gene is of great importance for ferroptosis [2], metabolic reprogramming [6] and cellular autophagy [15] in tumour cells. SCD (SCD1) is an endoplasmic reticulum (ER)-binding enzyme that catalyses the conversion of saturated fatty acids to monounsaturated fatty acids, and changes in the levels of both can alter cell membrane fluidity, leading to a range of cellular mechanisms associated with metabolism and carcinogenesis [15]. Studies reported that SCD is highly expressed in a variety of tumour tissues and can promote the proliferation and migration of tumour cells [6], which is closely related to tumourigenesis and progression, and is expected to become a new cervical cancer marker. Demonstrating the relationship between SCD and clinical features, the FIGO stage IV patients who developed distant metastases had the highest SCD expression (Fig. 1B). Likewise, in the other dataset (GSE44001), patients with advanced stages also had high expression (critical statistical significance). More importantly, there was also an association between high SCD expression and the depth of infiltration of tumour tissue. We then examined the prognostic significance of SCD on the prognosis of patients with cervical cancer, and the high-expression SCD group had shorter overall survival, disease-free survival intervals and progression-free survival compared to patients with low-expression SCD (Fig. 1C, D). As reported in the literature, a significant association of the ferroptosis pathway with high SCD expression was similarly demonstrated in the TCGA-CESC cohort using GSEA analysis (Fig. 1E).

2. **SCD1 is highly expressed in clinical cervical cancer tissues**,** especially in advanced tissue**.

**Fig. 2:**
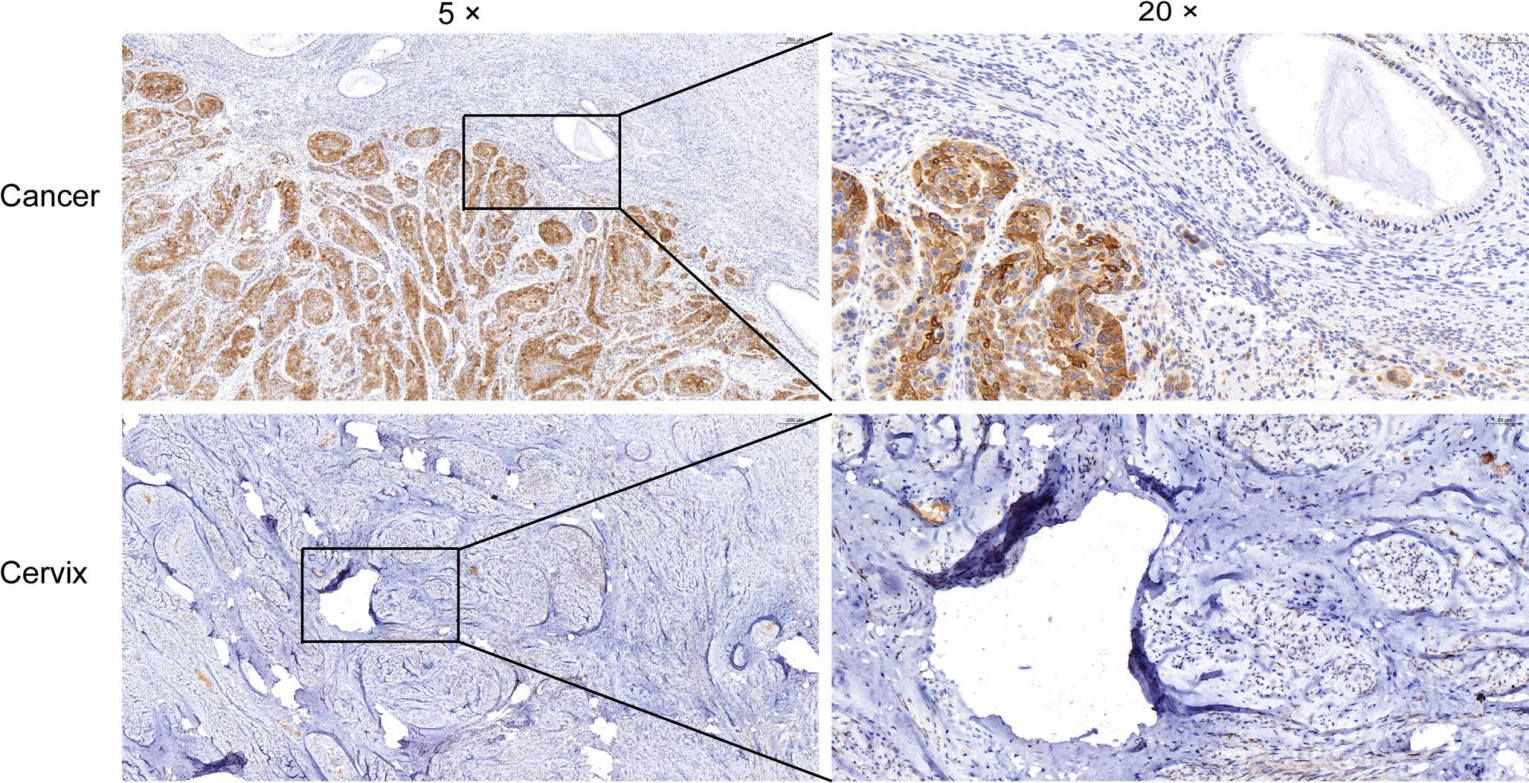
SCD1 is highly expressed in clinical cervical cancer tissues. Immunohistochemistry retrospectively analysed SCD1 expression in cervical cancer tissues and cervical tissues. Left:5 times. Scale bar: 200 μm. Right: 20 times. Scale bar: 50 μm

To verify the results of the pre-bioinformatics, we use immunohistochemistry retrospectively analysed the cervical tissues of 72 cases with cervical cancer (63 stage I-II, 9 stage III-IV) and 63 cervical tissues of cases who underwent total hysterectomy for benign uterine diseases during the same period in the Second Affiliated Hospital of Soochow University and the Liyang People’s Hospital of from December 2022 to November 2024. The results of the study group were analysed by immunohistochemical staining (IHC). The results showed that the expression of SCD1 in cervical cancer tissues was significantly higher than that in normal cervical tissues (Fig. 2). The basic information of the two groups of cases is shown in Table 1. The differences between the age, HPV infection positivity, and high expression of SCD1 in the study group were statistically significant, *p* < 0.0001. The difference between the groups with a history of combined medical and surgical diseases was statistically insignificant, *p*>0.05. There was no statistically significant difference between the fertility history groups, *p* > 0.05. The basic information between the groups of early and advanced cervical cancer cases is shown in Table 2. The results showed that the expression of SCD1 was statistically significant between the groups, *p* = 0.0467. The difference between the groups with positive HPV infection was significant, *p* < 0.0001. The difference between the groups with age, history of combined medical and surgical diseases, and history of childbearing was not statistically significant, *p* > 0.05.

**Table 1:** Basic information with cervical cancer cases and underwent total hysterectomy for benign diseases cases during the same period

Age (year)	Study group (*n* = 72)	Control group (*n* = 63)	χ^2^	*p*	OR	95% CI
	51.1 (33–82)	44.0 (35–63)	-	<0.0001	-	-
HPV infected			101.3	<0.0001	197.2	46.60–609.0
Positive (%)	68 (94.4%)	5 (7.9%)	-	-	-	-
Negtive (%)	4 (5.6%)	58 (92.1%)	-	-	-	-
Fertility history (> 2 pregnancies)	49 (68.1%)	37 (58.7%)	1.264	0.2858	1.497	0.7370–3.099
Combined medical and surgical diseases	50 (69.4%)	56 (88.9%)	7.532	0.3237	0.2841	0.1133–0.6956
High expression of SCD1	65 (90.3%)	1 (1.6%)	105.8	<0.0001	575.7	74.27–5845

**Table 2:** Basic information of cases in early and advanced cervical cancer cases

Age (year)	Early stage (*n* = 63)	Advanced stage (*n* = 9)	χ^2^	*p*	OR	95% CI
	52.37 (34–82)	58.56 (33–82)	-	0.2058	-	-
HPV infected			0.6050	<0.0001	0	0.000–8.292.000.292
Positive (%)	59 (93.7%)	9 (100%)	-	-	-	-
Negtive (%)	4 (6.3%)	0	-	-	-	-
Fertility history (> 2 pregnancies)	42 (66.7%)	7 (77.8%)	0.4472	0.5037	0.5714	0.1129–2.665
Combined medical and surgical diseases	44 (69.8%)	6 (66.7%)	0.0374	0.8466	1.158	0.2925–4.335
High expression of SCD1	43 (68.3%)	9 (100%)	3.956	0.0467	0	0.000–0.9740.000.9740

3. **Knockdown SCD1 inhibits proliferation of cervical cancer cells**.

**Fig. 3:**
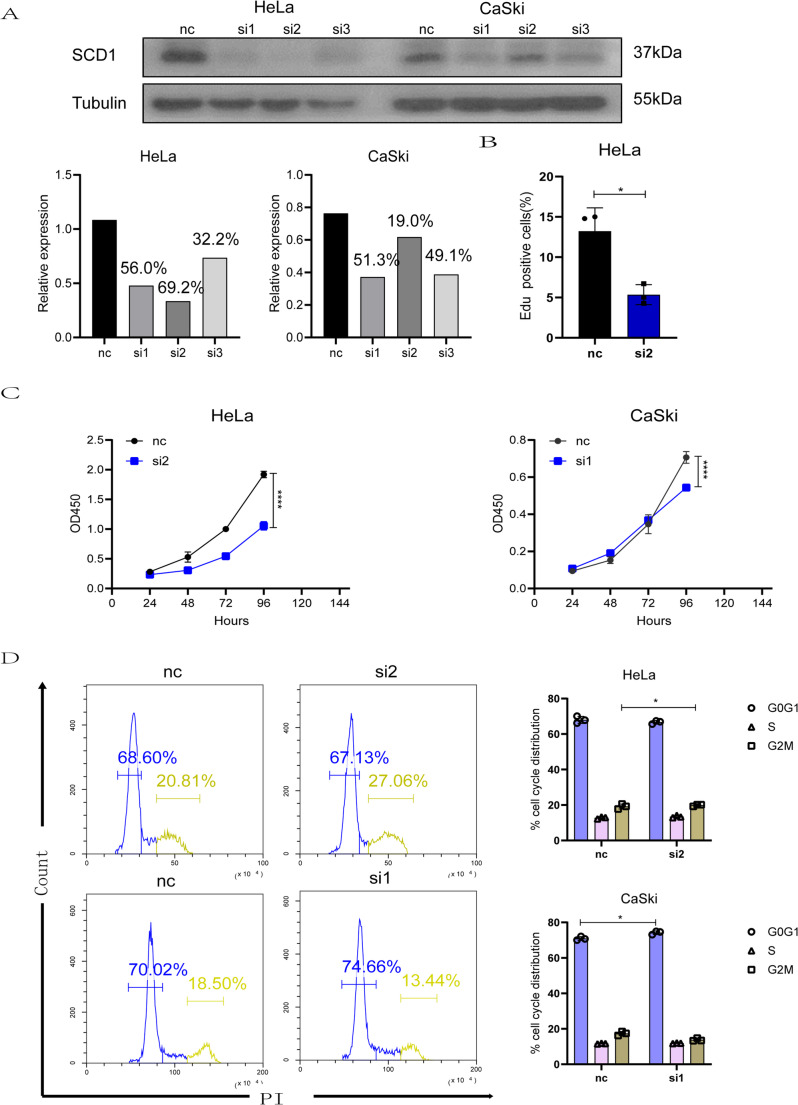
Knockdown SCD1 inhibits proliferation in cervical cancer cells. (**A**) Western blotting was used to detect the effect of SCD1 knockdown. (**B**) EdU proliferation assay was performed 48 h after the incubation. (**C**) Cell viability was measured by CCK-8 assay. The experiments were performed in duplicate for 24 h, 48 h, 72 h, and 96 h. (**D**) Cell cycle distribution in CaSki cells. The distribution of cell cycle was assessed by flow cytometry

In order to investigate the specific mechanism of SCD1 regulation in cervical cancer, we chose the adenocarcinomacell cacer cell line HeLa and squamous cell cacer cell line CaSki for cell experiments. Three different siRNAs were used to knock down SCD1, and WB was used to detect the effect of SCD1 knockdown. The results are shown in Fig. 3A, the knockdown efficiencies of si1, si2 and si3 were 56%, 69.2% and 32.2% in HeLa cells, and were 51.3%, 19% and 49.1% in CaSki cells, respectively. Hence si2RNA for HeLa versus si1RNA was selected for CaSki in the following experiments. As shown in Fig. 3B, SCD1 knockdown significantly reduced the percentage of EdU positive cells in HeLa cells. The effect on the proliferative capacity of cervical cancer cells after SCD1 knockdown were measured for their viability via the CCK8 assay. The results were shown in Fig. 3C, difference between groups ****p < 0.0001. To verify whether the inhibitory effect on proliferative capacity after knockdown of SCD1 was caused by cell cycle arrest, we detected the cell cycle by flow cytometry, and the results were shown in 3D: HeLa had G2/M phase arrest versus CaSki had G0/G1 phase arrest.

4. **Knockdown SCD1 induces ferroptosis and inhibits migration in cervical cancer cells**.

**Fig. 4:**
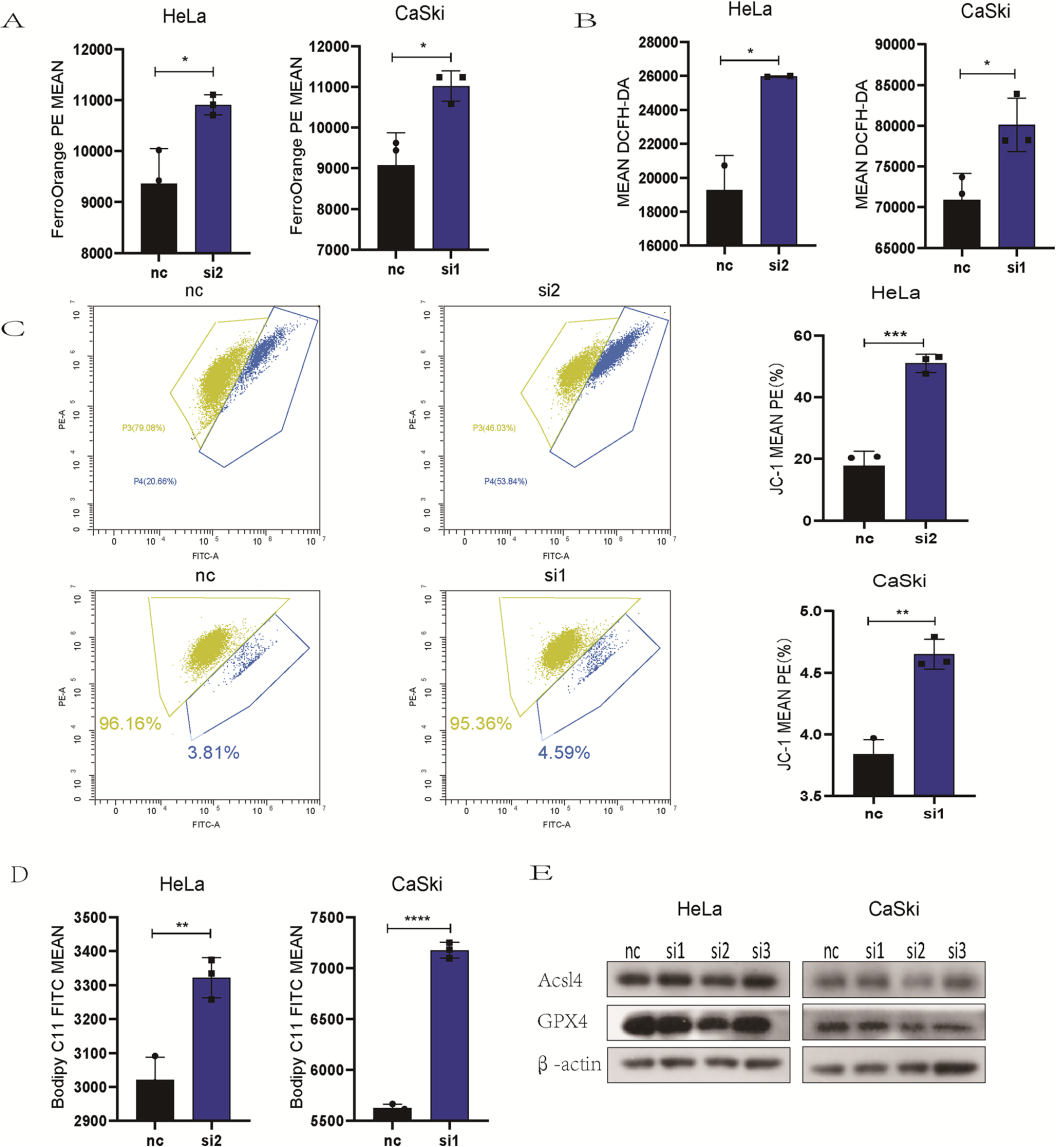
Knockdown of SCD1 induces ferroptosis in cervical cancer cells. (**A**) Bodipy C11 probe was used for measuring lipid per-oxidation by flow cytometry. Means and S.D.s of three repeats were shown. (**B**) ROS generation was measured using oxidation-sensitive fluorescent probe (DHE) by flow cytometry. Means and S.D.s of three repeats were shown. (**C**) Mitochondrial membrane potential was measured using JC-1 probe by flow cytometry. (**D**) Fe2+ generation was measured using fluorescent probe FerroOrange by flow cytometry. (**E**) Whole cell lysates were analyzed by immunoblotting with antibodies specific for Acsl4 and GPX4. β-actin was used as a loading control

**Fig. 5:**
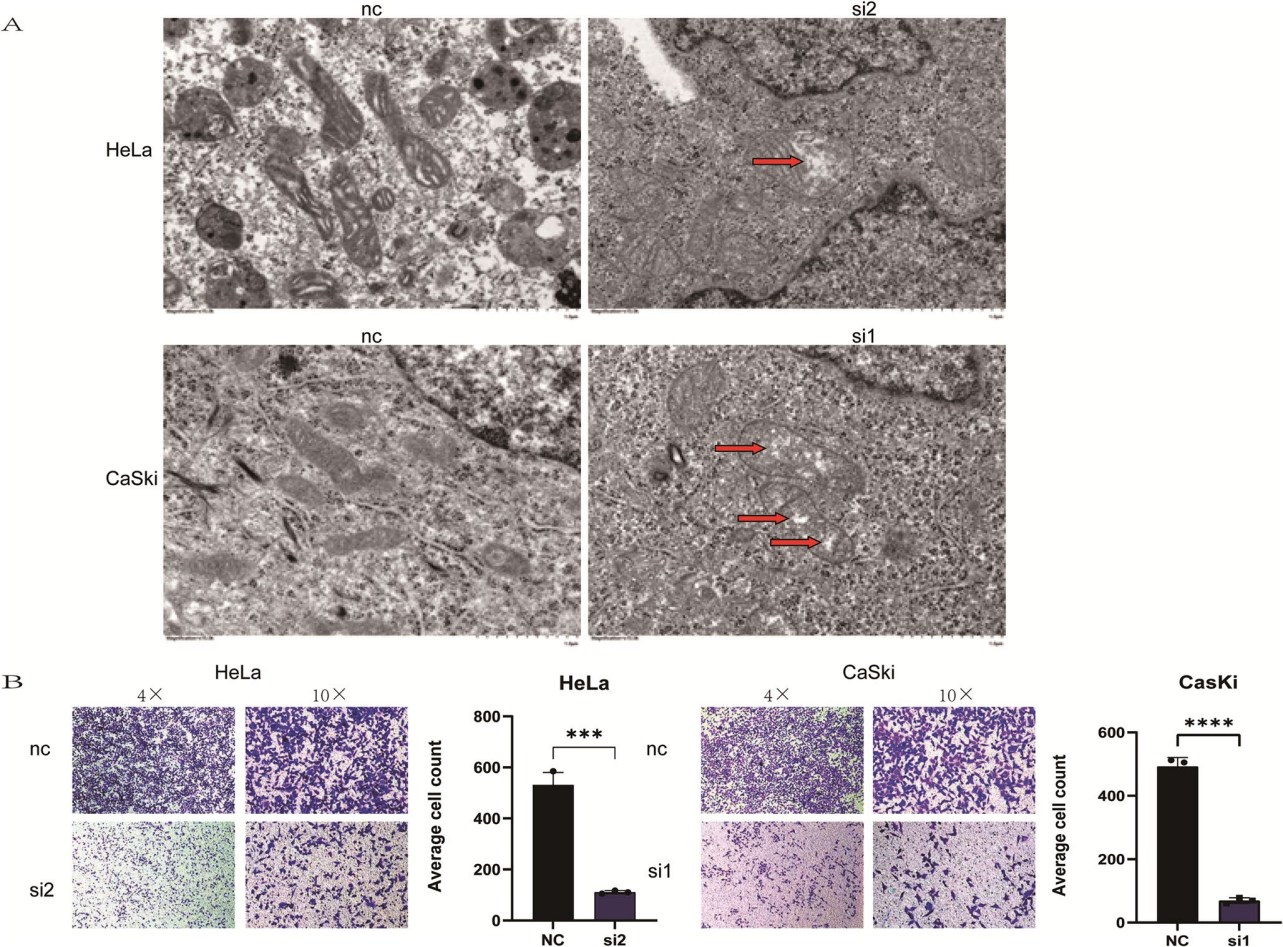
Knockdown SCD1 induces ferroptosis and inhibits migration in cervical cancer cells. (**A**) Transmission electron microscopy observes mitochondrial morphology after SCD1 knockdown (*n* = 3). Scale bar: 1 μm. (**B**) Migration ability was detected by Transwell assay (*n* = 3). 4× Scale bar: 200 μm. 10× Scale bar: 80 μm. The experiment was repeated 3 times and each contained at leas three biological replicates

To further explore the effects of SCD1 knockdown on cervical cancer cells, we detected intracellular Fe^2+^ content by flow cytometry, as shown in Fig. 4A: knockdown SCD1 significantly upregulated intracellular Fe^2+^ content in HeLa and CaSki cells. Next, we detected reactive oxygen species ROS with flow cytometry, DCFH-DA probe, as shown in Fig. 4B, ROS were significantly upregulated in HeLa and CaSki cells after SCD1 knockdown. We then detected mitochondrial membrane potential(MMP) by flow cytometry, as shown in Fig. 4C: MMP was significantly downregulated in HeLa and CaSki cells after SCD1 knockdown. We detected lipid peroxidation with BODIPY C11 probe, as shown in Fig. 4D, after SCD1 knockdown, lipid peroxidation was significantly upregulated in HeLa and CaSki cells. The expression levels of GPX4 and Acsl4, which are known to regulate ferroptosis (Fig. 4E), suggesting that the cancer cells underwent ferroptosis. We then used transmission electron microscopy to observe mitochondrial morphology after SCD1 knockdown and the results are shown in Fig. 5A: cell membrane rupture and exfoliation, membrane density increased, mitochondrial ridges decreased or disappeared, mitochondrial outer membrane rupture, mitochondrias became smaller and bilayer membrane densities increased. Immediately, we detected the migration ability by Transwell assay, as shown in 5B: the migration ability of cervical cancer cells were significantly inhibited after SCD1 knockdown. The difference between the groups was ****p < 0.0001.

5. **The relationship between SCD1 and CD8⁺ T cells is inverse in cervical cancer tissues**.

**Fig. 6:**
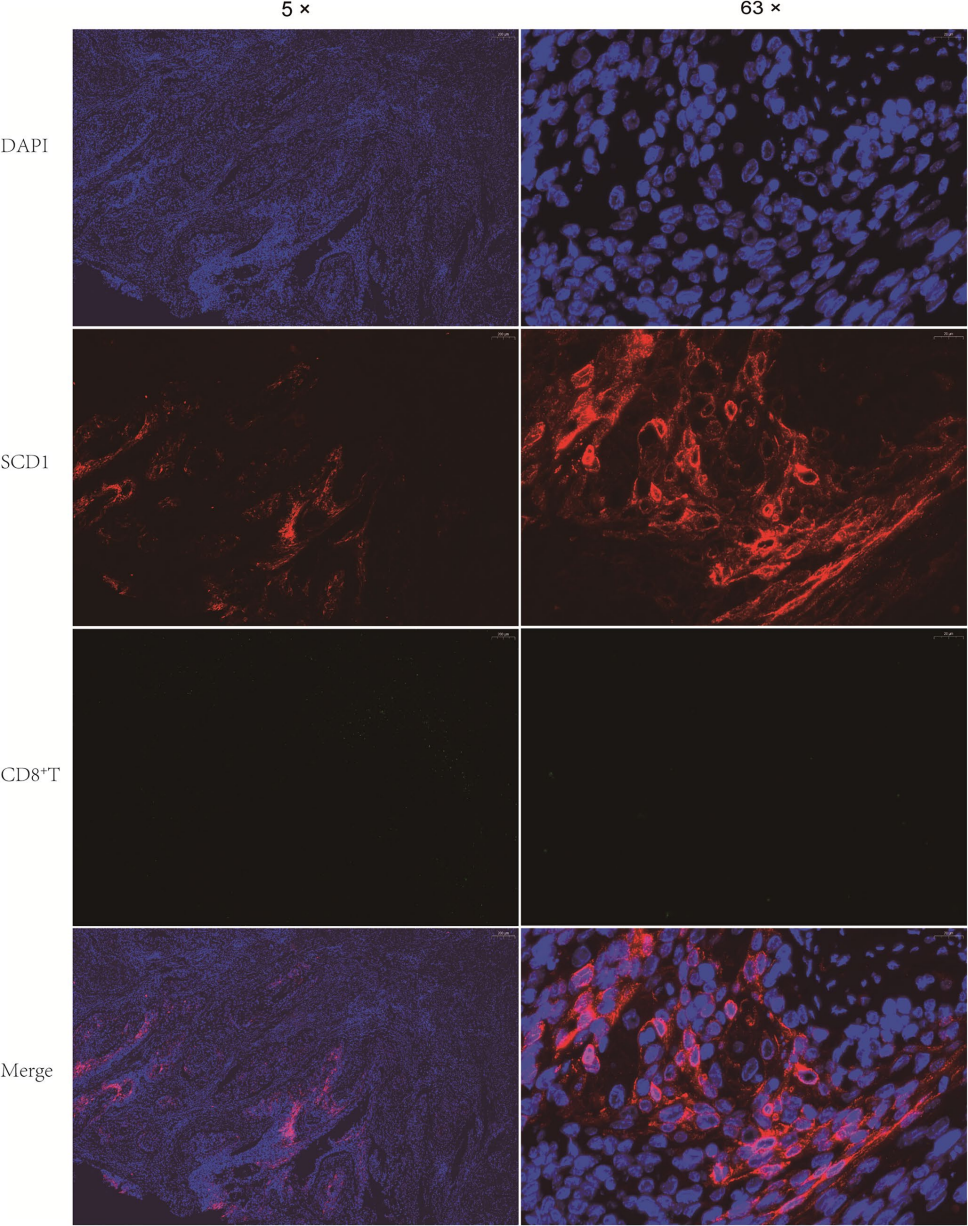
Heterogeneity of CD8^+^ T cell expression in cancer tissues and peripheral blood of cases with cervical cancer Immunofluorescence (IF) detection of SCD1/CD8^+^ T cells immunolocalisation in cervical cancer tissues (*n* = 72) showed high expression of SCD1 (SCD1, abcam, ab236868, ab19862, 1:100) in cancer tissues along with low expression of CD8^+^ T cell (CD8 + T, abcam, ab245118, ab237709, 1:100). 5×: Scale bar: 200 μm. 63×: Scale bar: 20 μm. SCD1: red fluorescent. CD8 + T: green fluorescent. DAPI (Beyotime, C1006) was used for the nuclear staining (blue fluorescent). The experiment was repeated at leas three biological replicates

Immunofluorescence (IF) detection of SCD1/CD8^+^ T cells immunolocalisation in cervical cancer tissues showed high expression of SCD1 in cancer tissues along with low expression of CD8^+^ T cells (Fig. 6).

6. **Heterogeneity of CD8**^**+**^ **T cell expression in cancer tissues and peripheral blood of cases with cervical cancer**.

The results of CD8^+^ T cell expression in 9 cancer tissues and 20 peripheral blood samples from cervical cancer patients are shown in Table 3.

**Table 3:** Heterogeneity of CD8 + T expression in cancer tissues and peripheral blood of cases with cervical cancer

CD8^+^T	Cancer tissue (*n* = 9)	Peripheral blood (*n* = 20)	χ^2^	*p*	OR	95% CI
			5.712	0.0169	0	0.000–0.6464.000.6464
Upregulate	0	6(29.4%)	-	-	-	-
Downregulate	9(100.0%)	7(35.3%)	-	-	-	-

## Discussion

In 2023, 13,960 new cases of cervical cancer were diagnosed in the US and an estimated 4,310 people died from the disease [16]. 85% of cases occured in developing countries, and cervical cancer was the leading cause of cancer death in people assigned female at birth [17, 18]. More than 99% of cervical cancers are associated with certain types of HPV, known as high-risk types [19]. Squamous cell carcinoma (SCC), adenocarcinoma (AC) and adenosquamous carcinoma (ASC) are the three most common histologies of cervical cancer [20]. Accounting for approximately 80% of all cervical cancers [21]. Currently, there is no difference in treatment between the SCC and AC/ASC subtypes of cervical cancer, although the clinical features and prognosis of the disease vary widely between these subtypes. Regardless of cancer subtype and HPV infection status, primary treatment with curative intent cervical cancer patients typically includes surgery, radiotherapy, or combination of these treatments; the choice varies depending on cancer stage. The NCCN guideline insights highlight the latest updates in systemic treatment options for cervical cancer [22]. Even so, there are still many patients whose prognosis is compromised by drug resistance and recurrence. Therefore, we need to take advantage of advances in the understanding pathophysiology of cervical cancer to explore new therapeutic targets to improve prognosis.

Upregulation of stearoyl coenzyme A desaturase (SCD), which synthesises monounsaturated fatty acids, is required for cancer cell proliferation in a lipid-depleted environment [23]. However, the role of SCD1 in cervical cancer and the mechanisms involved remain unclear, and there is only one report of changes in SCD levels in an invasive metastatic human cervical cancer HTB-34 cell line after metformin treatment [24]. In this study, the function of SCD1 in cervical cancer was analysed in detail. Firstly, we have previously learned from bioinformatic search databases that members of the SCD protein family are highly expressed oncogenes in cervical cancer and are an indicator of prognosis in cervical cancer patients. It was also shown that the ferroptosis pathway was significantly associated with high SCD expression. We then confirmed the high expression of SCD1 in cervical cancer tissues using immunohistochemistry. We also investigated the effect of SCD1 on the malignant biological behaviour of cancer cells using the cervical cancer HeLa and CaSki. The results showed that knockdown of SCD1 inhibited the proliferation and migration ability of cervical cancer cells. Most encouragingly, knockdown of SCD1 induces ferroptosis in cervical cancer cells. Ferroptosis is a novel mode of programmed cell death dependent on intracellular iron but not other metals, driven by lipid peroxidation and morphologically, biochemically and genetically distinct from apoptosis, necrosis and autophagy [16]. There are three main areas of research that converge to provide a fundamental understanding of what we now call the field of ferroptosis, (1) metabolic mechanisms, (2) control of reactive oxygen species (ROS), (3) regulation of iron [25]. Mitochondria are the primary site of ROS production and can act as initiators and amplifiers of cysteine starvation-induced ferroptosis [26]. Divalent iron ions are maintained in the cell in the form of unstable iron pools bound to low molecular weight compounds, including GSH [27]. Inhibition of the mitochondrial electron transport chain also attenuates Cys starvation-induced ferroptosis, as does mitochondrial depletion. The role of the electron transport chain may be due to the generation of superoxide and electron leakage from H_2_O_2_, which can then react with Fe^2+^ to drive Fenton chemistry and lipid peroxidation [28]. The present study revealed that after knockdown of SCD1, cervical cancer cells showed accumulation of ferrous ions, lipid peroxidation, up-regulation of reactive oxygen species, down-regulation of cellular membrane potential, fracture and vesiculation of cellular membranes as observed by transmission electron microscopy, Mitochondria became smaller, membrane density increased, mitochondrial ridges were reduced or disappeared and the outer mitochondrial membranes were fractured, and the nuclei of the cells were of normal size but lacked chromatin condensation, consistent with the previous study.

Enzyme long-chain family member 4 (ACSL4) and lysophosphatidylcholine acyltransferase 3 (LPCAT3) produce lipid drivers of ferroptosis [25]. ACSL4 is the first pro-ferroptosis gene product identified. Its overexpression is sensitive to ferroptosis and may be more analogous to caspase-3 [25], the executor of apoptosis, which inhibits lipid ROS accumulation. Degradation of GPX4 also promotes ferroptosis [29]. Depletion of GSH not only inactivates GPX4 but also mobilises Fe^2+^ for Fenton chemistry, which promotes the propagation of lipid peroxides and ultimately ferroptosis [30]. In the present study, we demonstrated that the expression of ACSL4 was significantly up-regulated in cervical cancer cells after knockdown of SCD1, and at the same time the expression of GPX4 was significantly down-regulated, which is consistent with the results previously reported in the literature.

In addition to developing potent and selective compounds and biologics that can modulate ferroptosis by specific mechanisms, it is important to expand the toolset for reliable detection of ferroptosis and other modes of cell death. Oxidation of the C11-BODIPY fluorescent probe and reactivity with antibodies that detect adducts formed by lipid peroxidation products. In the present study, the C11-BODIPY fluorescent probe was used to detect a significant up-regulation of lipid peroxidation in cervical cancer cells after SCD1 knockdown. Our results have stimulated new ideas and further exploration for the treatment of cervical cancer. Given the large number of recent studies showing that ferroptosis is associated with the development and progression of diseases such as tumours, there may be an opportunity to treat these diseases by modulating ferroptosis.

Numerous immune functions depend on the proper efficiency of mitochondrial metabolism in immune cells [31]. Dysregulation of mitochondrial dynamics contributes to the development and progression of various cancers, affecting tumour cell proliferation, metastasis, drug resistance and the tumour microenvironment (TME), suggesting that the study of mitochondrial dynamics may be a promising approach for anti-tumour therapy. Uncontrolled cell growth, disruption of cell cycle regulation, and aberrant cell programme death characterise cancer [32]. During the process of ferroptsis, intracellular iron ions may cause mitochondrial damage and trigger the process of mitochondrial autophagy to help the cell remove damaged mitochondria and protect the cell from iron-induced damage. Recent studies have shown that mitochondrial dynamics have a profound effect on immune monitoring within the TME. Infiltrating T-lymphocytes (TEL) usually show exhaustion and their fate is thought to be regulated by mitochondrial dynamics [33]. Mitochondrial dynamics and metabolites are key to optimising the anti-tumour function of T cells [34]. Cancer cells can evade CD8^+^ T cell attack through various strategies, such as decreasing the expression of tumour-specific antigens, increasing the infiltration of immunosuppressive cells, blocking the activation of CD8^+^ T cells, and inducing CD8^+^ T cell depletion and death [35–37]. CD8^+^ T cells are the most potent immune cells in anticancer immunity and can directly kill cancer cells in several ways. Iron death plays a crucial role in CD8^+^ T cell-mediated anti-tumour immunity. However, CD8^+^ T cells are also vulnerable in TME: Cancer cells up-regulate molecules such as PD-L1 and Fas-L, leading to CD8^+^ T cell dysfunction and depletion, and promoting immune escape from cancer cells [38]. Novel evidence also suggests that cancer cells induce iron death of CD8^+^ T cells by interfering with TME, weakening their anti-cancer immune function [39]. Recent studies have shown that CD8^+^ T cells are more sensitive to ferroptosis than cancer cells and are susceptible to spontaneous ferroptosis influenced by TME, thus depriving CD8^+^ T cells of their chance of survival [39]. This leads to reduced abundance and induced dysfunction of CD8^+^ T cells within the tumour and promotes immune escape of cancer cells. Deficiency of ACSL4 protects CD8^+^ T cells from the threat of high-dose RSL3-induced ferroptosis, but also leads to functional defects in CD8^+^ T cells, enabling cancer cells to evade specific killing by CD8^+^ T cells [40]. Thus, ACSL4, a key enzyme that regulates lipid peroxidation during cellular ferroptosis, is required to maintain CD8^+^ T cell function [39]. IFNγ is one of the effectors secreted by activated CD8^+^ T cells [41] and exerts its function by binding to INFγ on cancer cells. In the presence of IFNγ, activation of JAK1/2 and phosphorylation of STAT1 increased IRF1 expression on cancer cells [42]. IRF1 serves as a transcriptional activator at the ACSL4 promoter [43]. Consequently, IFNγ in combination with AA has been suggested to be an endogenous trigger for ACSL4-mediated ferroptosis in cancer cells [44]. Kong [45] et al. demonstrated that IFNγ treatment induced cancer cell ferroptosis via the STAT1/IRF1/ACSL4 axis in hepatocellular carcinoma, and they also found that the combination of IFNγ and Erastin enhanced mitochondrial oxidation and loss of mitochondrial membrane potential (MMP), which further triggered ferroptosis. IFNγ also promotes lipid peroxidation via ACSL4 through the use of fatty acids and leads to selective enrichment of PUFA to induce cellular ferroptosis [46]. In summary, activated CD8^+^ T cells secrete IFN-γ and act on key targets of ferroptosis in cancer cells. This results in a reduced oxidative buffering capacity of GPX4 and other GSH-dependent enzymes.

Research on the impact of SCD1 and CD8 T cells in vivo remains limited. SCD1 inhibitor was found to directly stimulate dendritic cells (DCs) and CD8^+^ T cells [47]. SCD1 expressed in cancer cells and immune cells causes immunoresistant conditions, and its inhibition augments antitumor CD8^+^ T cells [8]. Induction of SCD1 by lactic acid-induced endothelial cell-specific molecule 1 (ESM1) can impede the CD8^+^ T-cell response against tumors and promote resistance to cisplatin by activating the Wnt/β-catenin pathway in ovarian cancer [48]. Inactivation of Scd-1 triggers the specialization of CD8^+^ T cells into the Teff subset, enhancing the effector function and mitochondrial metabolism of Teff cells [49]. Based on the above research background, this suggests that SCD1 under investigation may modulate the Wnt/β-catenin signalling pathway, interfere with fatty acid conversion, recruit chemokines, and reduce the recruitment and activation of CD8^+^ T cells, thereby attenuating antitumour immune responses. Regrettably, due to funding and time constraints, we can’t strengthen this aspect. Definitely, the sample size for CD8⁺ T cell tissue analysis (*n* = 9) is very small, our results were preliminary and needed further validation. We will explore whether SCD1 acts through the PI3K/AKT/mTOR pathway. Subsequent studies should expand sample sizes to obtain more conclusive evidence. Probably, co-culture assays or cytokine researches may further elucidate the underlying mechanisms of interaction between SCD1 and CD8^+^ T cells. We wish our finding could provide a new potential method to treat clinical cervical cancer.

## Electronic Supplementary Material

Below is the link to the electronic supplementary material.

Supplementary file 1 (PNG 828 KB)

High Resolution Image (TIF 4.25 MB)

## Data Availability

The data that support the findings of this study are available from the corresponding author, Weipei Zhu (zwp333xx@126.com), upon reasonable request.

## References

[1] Kalafati E, Drakopoulou E, Anagnou NP, Pappa KI. Developing oncolytic viruses for the treatment of cervical cancer. Cells. 2023;12(14):1838. 37508503 10.3390/cells12141838 PMC10377776

[2] Hu Z, et al. Genome-wide profiling of HPV integration in cervical cancer identifies clustered genomic hot spots and a potential microhomology-mediated integration mechanism. Nat Genet. 2015;47(2):158–63. 25581428 10.1038/ng.3178

[3] Sen U, Coleman C, Sen T. Stearoyl coenzyme A desaturase-1: multitasker in cancer, metabolism, and ferroptosis. Trends Cancer. 2023;9(6):480–9. 37029018 10.1016/j.trecan.2023.03.003

[4] Carbone M, Melino G. Stearoyl CoA desaturase regulates ferroptosis in ovarian cancer offering new therapeutic perspectives. Cancer Res. 2019;79(20):5149–50. 31615810 10.1158/0008-5472.CAN-19-2453

[5] Dixon SJ, et al. Ferroptosis: an iron-dependent form of nonapoptotic cell death. Cell. 2012;149(5):1060–72. 22632970 10.1016/j.cell.2012.03.042 PMC3367386

[6] Yu Y, Kim H, Choi S, Yu J, Lee JY, Lee H, Yoon S, Kim WY. Targeting a lipid desaturation enzyme, SCD1, selectively eliminates colon cancer stem cells through the suppression of Wnt and NOTCH signaling. Cells. 2021;10(1):106. . 10.3390/cells10010106 33430034 10.3390/cells10010106 PMC7826607

[7] Han C, et al. IGF2BP3 enhances lipid metabolism in cervical cancer by upregulating the expression of SCD. Cell Death Dis. 2024;15(2):138. 38355626 10.1038/s41419-024-06520-0 PMC10867090

[8] Gong Y, et al. Peptostreptococcus anaerobius promotes cervical cancer angiogenesis by upregulating SCD to activate ERK pathway. Am J Cancer Res. 2025;15(7):3236–44. 40814384 10.62347/DYUN7645 PMC12344179

[9] Wang Y, Patti GJ. The warburg effect: a signature of mitochondrial overload. Trends Cell Biol. 2023;33(12):1014–20. 37117116 10.1016/j.tcb.2023.03.013 PMC10600323

[10] Greenwood HE, Witney TH. Latest advances in imaging oxidative stress in cancer. J Nucl Med. 2021;62(11):1506–10. 34353871 10.2967/jnumed.120.256974 PMC7611938

[11] Ladner C, et al. Effect of etoposide (VP16-213) on lipid peroxidation and antioxidant status in a high-dose radiochemotherapy regimen. Cancer Chemother Pharmacol. 1989;25(3):210–2. 2513140 10.1007/BF00689585

[12] Tauriello DVF, et al. TGFbeta drives immune evasion in genetically reconstituted colon cancer metastasis. Nature. 2018;554(7693):538–43. 29443964 10.1038/nature25492

[13] Monaco G, et al. RNA-Seq signatures normalized by mRNA abundance allow absolute deconvolution of human immune cell types. Cell Rep. 2019;26(6):1627–e16407. 30726743 10.1016/j.celrep.2019.01.041 PMC6367568

[14] Zhang N, Bevan MJ. CD8(+) T cells: foot soldiers of the immune system. Immunity. 2011;35(2):161–8. 21867926 10.1016/j.immuni.2011.07.010 PMC3303224

[15] Wohlhieter CA, et al. Concurrent mutations in STK11 and KEAP1 promote ferroptosis protection and SCD1 dependence in lung cancer. Cell Rep. 2020;33(9):108444. 33264619 10.1016/j.celrep.2020.108444 PMC7722473

[16] Siegel RL, et al. Cancer statistics, 2023. CA Cancer J Clin. 2023;73(1):17–48. 36633525 10.3322/caac.21763

[17] Parkin DM, et al. Global cancer statistics, 2002. CA Cancer J Clin. 2005;55(2):74–108. 15761078 10.3322/canjclin.55.2.74

[18] Kamangar F, Dores GM, Anderson WF. Patterns of cancer incidence, mortality, and prevalence across five continents: defining priorities to reduce cancer disparities in different geographic regions of the world. J Clin Oncol. 2006;24(14):2137–50. 16682732 10.1200/JCO.2005.05.2308

[19] Yuan Y, et al. HPV post-infection microenvironment and cervical cancer. Cancer Lett. 2021;497:243–54. 33122098 10.1016/j.canlet.2020.10.034

[20] Landrigan PJ, et al. The Minderoo-Monaco Commission on Plastics and Human Health. Ann Glob Health. 2023;89(1):23. 36969097 10.5334/aogh.4056 PMC10038118

[21] Jemal A, et al. Global cancer statistics. CA Cancer J Clin. 2011;61(2):69–90. 21296855 10.3322/caac.20107

[22] Abu-Rustum NR, et al. NCCN guidelines insights: cervical cancer, version 1.2024. J Natl Compr Canc Netw. 2023;21(12):1224–33. 38081139 10.6004/jnccn.2023.0062

[23] Lien EC, et al. Low glycaemic diets alter lipid metabolism to influence tumour growth. Nature. 2021;599(7884):302–7. 34671163 10.1038/s41586-021-04049-2 PMC8628459

[24] Ascenzi F, et al. SCD1, autophagy and cancer: implications for therapy. J Exp Clin Cancer Res. 2021;40(1):265. 34429143 10.1186/s13046-021-02067-6 PMC8383407

[25] Stockwell BR. Ferroptosis turns 10: Emerging mechanisms, physiological functions, and therapeutic applications. Cell. 2022;185(14):2401–21. 35803244 10.1016/j.cell.2022.06.003 PMC9273022

[26] Mao C, et al. DHODH-mediated ferroptosis defence is a targetable vulnerability in cancer. Nature. 2021;593(7860):586–90. 33981038 10.1038/s41586-021-03539-7 PMC8895686

[27] Patel SJ, et al. A PCBP1-BolA2 chaperone complex delivers iron for cytosolic [2Fe-2S] cluster assembly. Nat Chem Biol. 2019;15(9):872–81. 31406370 10.1038/s41589-019-0330-6 PMC6702080

[28] Gao M, et al. Role of mitochondria in ferroptosis. Mol Cell. 2019;73(2):354–e3633. 30581146 10.1016/j.molcel.2018.10.042 PMC6338496

[29] Shimada K, et al. Global survey of cell death mechanisms reveals metabolic regulation of ferroptosis. Nat Chem Biol. 2016;12(7):497–503. 27159577 10.1038/nchembio.2079 PMC4920070

[30] Doll S, et al. ACSL4 dictates ferroptosis sensitivity by shaping cellular lipid composition. Nat Chem Biol. 2017;13(1):91–8. 27842070 10.1038/nchembio.2239 PMC5610546

[31] Breda CNS, et al. Mitochondria as central hub of the immune system. Redox Biol. 2019;26:101255. 31247505 10.1016/j.redox.2019.101255 PMC6598836

[32] Hanahan D, Weinberg RA. Hallmarks of cancer: the next generation. Cell. 2011;144(5):646–74. 21376230 10.1016/j.cell.2011.02.013

[33] Buck MD, et al. Mitochondrial dynamics controls T cell fate through metabolic programming. Cell. 2016;166(1):63–76. 27293185 10.1016/j.cell.2016.05.035 PMC4974356

[34] Liu YN, et al. Hypoxia induces mitochondrial defect that promotes T cell exhaustion in tumor microenvironment through MYC-regulated pathways. Front Immunol. 2020;11:p1906. 10.3389/fimmu.2020.01906 PMC7472844 32973789

[35] Farhood B, Najafi M, Mortezaee K. CD8(+) cytotoxic T lymphocytes in cancer immunotherapy: a review. J Cell Physiol. 2019;234(6):8509–21. 30520029 10.1002/jcp.27782

[36] Marcovecchio PM, Thomas G, Salek-Ardakani S. CXCL9-expressing tumor-associated macrophages: new players in the fight against cancer. J Immunother Cancer. 2021;9(2):e002045. . 10.1136/jitc-2020-002045 33637602 10.1136/jitc-2020-002045 PMC7919587

[37] Kumar S, et al. Tumor-infiltrating CD8(+) T cell antitumor efficacy and exhaustion: molecular insights. Drug Discov Today. 2021;26(4):951–67. 33450394 10.1016/j.drudis.2021.01.002 PMC8131230

[38] Ge W, et al. PLA2G2A(+) cancer-associated fibroblasts mediate pancreatic cancer immune escape via impeding antitumor immune response of CD8(+) cytotoxic T cells. Cancer Lett. 2023;558:216095. 36796670 10.1016/j.canlet.2023.216095

[39] Drijvers JM, et al. Pharmacologic screening identifies metabolic vulnerabilities of CD8(+) T cells. Cancer Immunol Res. 2021;9(2):184–99. 33277233 10.1158/2326-6066.CIR-20-0384 PMC7864883

[40] Shan G, et al. Resveratrol improves the cytotoxic effect of CD8 + T cells in the tumor microenvironment by regulating HMMR/Ferroptosis in lung squamous cell carcinoma. J Pharm Biomed Anal. 2023;229:115346. 37001272 10.1016/j.jpba.2023.115346

[41] Luu M, et al. Microbial short-chain fatty acids modulate CD8(+) T cell responses and improve adoptive immunotherapy for cancer. Nat Commun. 2021;12(1):4077. 34210970 10.1038/s41467-021-24331-1 PMC8249424

[42] Wei J, et al. Parafibromin is a component of IFN-gamma-triggered signaling pathways that facilitates JAK1/2-mediated tyrosine phosphorylation of STAT1. J Immunol. 2015;195(6):2870–8. 26232434 10.4049/jimmunol.1501111

[43] Kong P, et al. Ferroptosis triggered by STAT1- IRF1-ACSL4 pathway was involved in radiation-induced intestinal injury. Redox Biol. 2023;66:102857. 37611494 10.1016/j.redox.2023.102857 PMC10466894

[44] Liao P, et al. CD8(+) T cells and fatty acids orchestrate tumor ferroptosis and immunity via ACSL4. Cancer Cell. 2022;40(4):365–e3786. 35216678 10.1016/j.ccell.2022.02.003 PMC9007863

[45] Kong R, et al. IFNgamma-mediated repression of system xc(-) drives vulnerability to induced ferroptosis in hepatocellular carcinoma cells. J Leukoc Biol. 2021;110(2):301–14. 34318944 10.1002/JLB.3MA1220-815RRR

[46] Friedmann Angeli JP, Xavier da TN, Silva, Schilling B. CD8(+) T cells PUF(A)ing the flames of cancer ferroptotic cell death. Cancer Cell. 2022;40(4):346–8. 35334204 10.1016/j.ccell.2022.03.003

[47] KatohY,et al. Inhibition of stearoyl-CoA desaturase 1 (SCD1) enhances the antitumor T cell response through regulating β-catenin signaling in cancer cells and ER stress in T cells and synergizes with anti-PD-1 antibody. J Immunother Cancer. 2022;10(7):e004616. . 10.1136/jitc-2022-004616 35793868 10.1136/jitc-2022-004616 PMC9260842

[48] Fan Z, et al. Lactate drives the ESM1-SCD1 axis to inhibit the antitumor CD8(+) T-cell response by activating the Wnt/beta-catenin pathway in ovarian cancer cells and inducing cisplatin resistance. Int Immunopharmacol. 2024;137:112461. 38897128 10.1016/j.intimp.2024.112461

[49] Lin Y, et al. Scd-1 deficiency promotes the differentiation of CD8(+) T effector. Front Cell Infect Microbiol. 2024;14:1325390. 38379772 10.3389/fcimb.2024.1325390 PMC10876803

