# Supplementary figures and images for: CD8+ T Cells in Association with SCD1 Regulate Cervical Cancer and Tumour Microenvironment Correlation Research
Source: Reprod Sci. 2026 Apr 22;33(7):1338–52. doi: 10.1007/s43032-026-02070-2 (PMC13421197; doi:10.1007/s43032-026-02070-2)

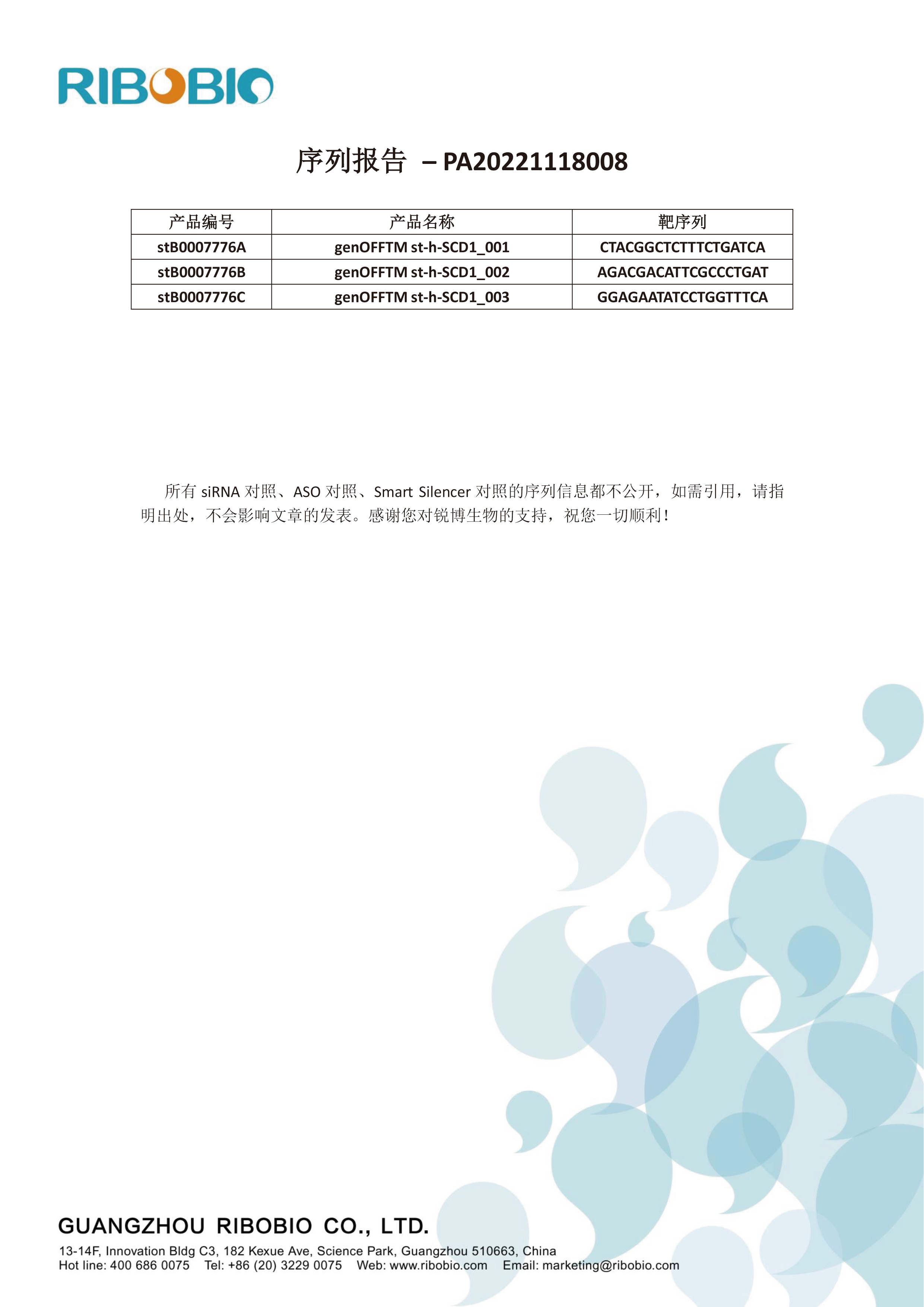

Supplement: Supplementary file 1 — (PNG 828 KB) [file 43032_2026_2070_Fig7_ESM.png]

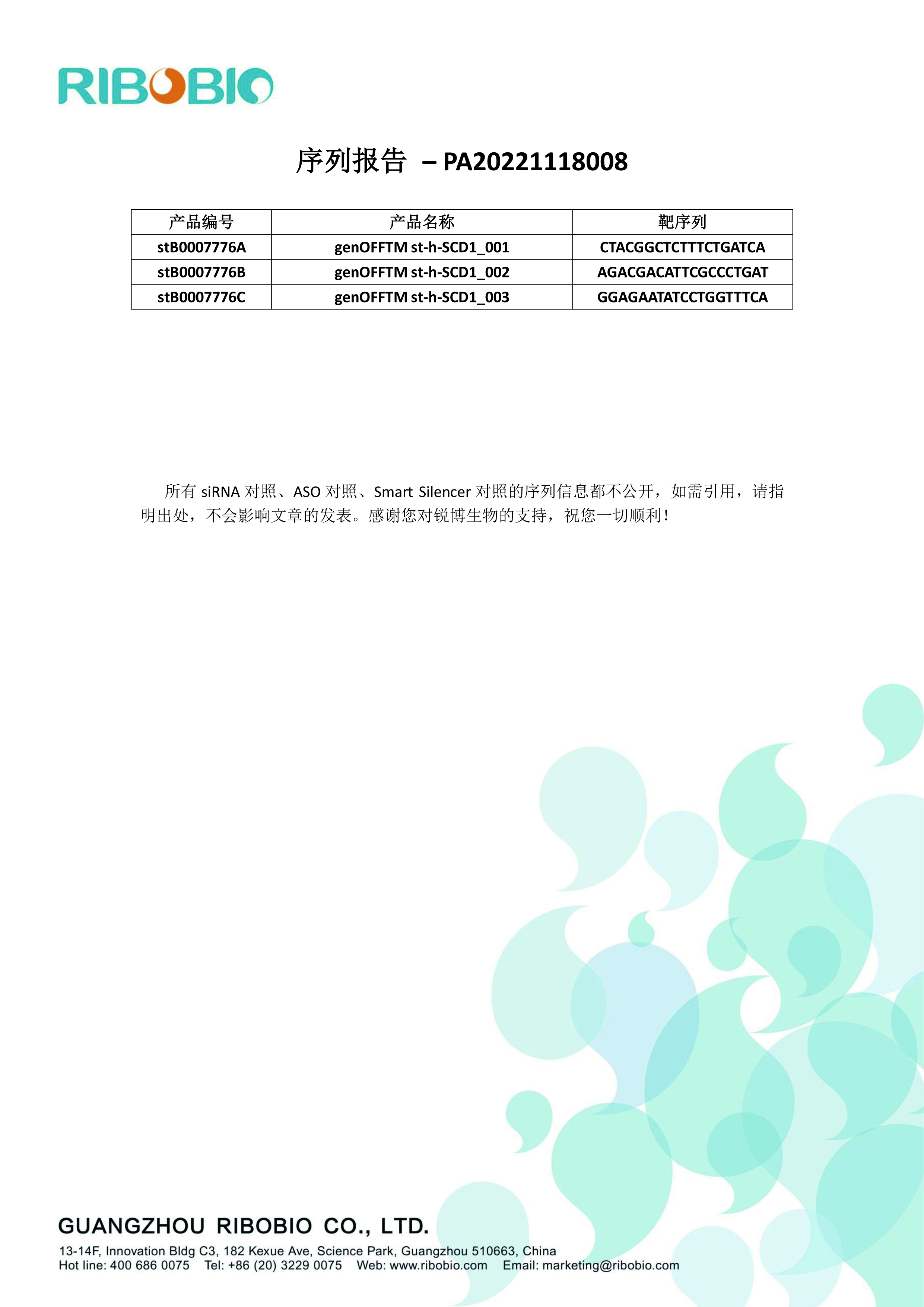

Supplement: Supplementary file 2 — High Resolution Image (TIF 4.25 MB) [file 43032_2026_2070_MOESM1_ESM.tif]
